# Effective Components of *Panax quinquefolius* and *Corydalis tuber* Protect Myocardium through Attenuating Oxidative Stress and Endoplasmic Reticulum Stress

**DOI:** 10.1155/2013/482318

**Published:** 2013-06-24

**Authors:** Mei Xue, Meilin Liu, Xinyuan Zhu, Lin Yang, Yu Miao, Dazhuo Shi, Huijun Yin

**Affiliations:** ^1^Cardiovascular Center, Xiyuan Hospital, China Academy of Chinese Medical Sciences, Beijing 100091, China; ^2^Department of Geriatric, Peking University First Hospital, Beijing 100034, China

## Abstract

Both oxidative stress and endoplasmic reticulum stress (ERS) have been implicated in carcinogenesis and neurological diseases, while there are few reports about the mechanisms of them in the progression of acute myocardial infarction (AMI). This study examined oxidative stress and ERS in a rat model of AMI and evaluated their role in therapy by metoprolol and effective components of *Panax quinquefolius* and *Corydalis tuber* (EPC). In the present study a rat model of AMI was established by ligation of the left anterior descending coronary artery. After oral administration of metoprolol or low-to-high doses of EPC for 2 weeks, serum malondialdehyde (MDA), superoxide dismutase (SOD), and 8-iso-prostaglandin F2**α** (8-iso-PGF2**α**) were detected using enzyme-linked immunosorbent assay (ELISA). Quantitative real-time PCR and Western blotting were used to examine mRNA and protein expressions of the hallmarks of ERS-glucose-regulated protein-78 (GRP78) and CCAAT/enhancer-binding protein homologous protein (CHOP). We confirmed that both metoprolol and moderate-to-high dose of EPC decreased 8-iso-PGF2**α** serum level and downregulated the mRNA and protein expressions of GRP78 and CHOP in myocardium, while EPC also increased SOD serum level. These results indicated that metoprolol and EPC protect the myocardium by attenuating oxidative stress and ERS induced by myocardial infarction, highlighting the ERS pathways as potential therapeutic targets for AMI.

## 1. Introduction

Acute myocardial infarction (AMI) is a severe stress condition that causes extensive biochemical changes, which is associated with increasing production of reactive oxygen species (ROS) [1]. The imbalance between ROS production and antioxidant defenses leads to the condition known as oxidative stress. Detrimental effects of ROS are clearly demonstrated by the findings that in transgenic mice in which an antioxidant protein, superoxide dismutase (SOD), is overexpressed, infarct size is markedly reduced [2, 3]. There is a growing body of evidence which indicates that oxidative stress plays an important role in the initiation and progression of myocardial infarction (MI) [4–7]. 

**Figure 1:**
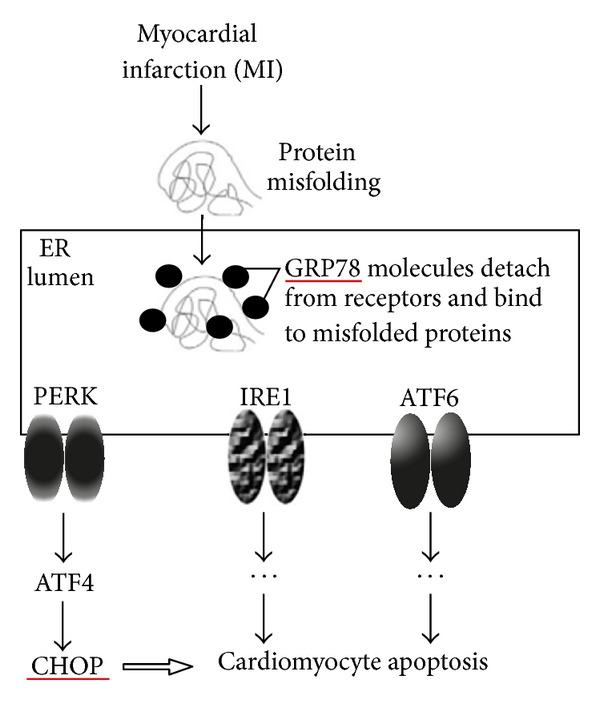
ERS during MI [11].

The endoplasmic reticulum (ER) is a multifunctional intracellular organelle responsible for the synthesis and folding of proteins as well as calcium storage and signaling. Various stimuli, such as ischemia, hypoxia, oxidative stress, and inflammatory factors, have been suggested to triggering ER dysfunction, which are designated as ER stress (ERS) [8, 9]. Cells alleviate ERS through the unfolded protein response (UPR). The upregulation of ER chaperones, such as the glucose-regulated protein-78 (GRP78), contributes to the repair of unfolded proteins. However, if stress is sustained, the UPR causes cell death by transcriptional induction of CCAAT/enhancer-binding protein homologous protein (CHOP), the caspase-12 dependent pathway, and activation of the c-Jun NH_2_-terminal kinase 1 (JNK1) dependent pathway [10]. Recently, Mitra et al. [11] reported that GRP78, as an ER-resident protein, assisting in protein folding and the most important upstream regulator of the UPR, was exclusively upregulated during MI. Exclusive upregulation of CHOP in MI hearts and nuclear translocation of CHOP in the hypoxic cardiomyocytes signifies induction of ERS-mediated apoptosis (Figure 1) [11]. Further, some data suggest that oxidative stress and ERS reinforce each other in thymic lymphomagenesis and sporadic amyotrophic lateral sclerosis [12–14], while there are very few reports about the mechanisms of them in the progression of MI.

The extracts of *Panax quinquefolius* and *Corydalis tuber* (EPC), composed of *Panax quinquefolius* saponins and tetrahydropalmatine mainly, showed good effects for the treatment of ischemic cardiovascular diseases in clinic. *Panax quinquefolius* saponins and tetrahydropalmatine have been shown to have protective effects against oxidative stress [15–17]. Recent study demonstrated that *Panax quinquefolius *saponins can also reduce myocardial hypoxia-reoxygenation injury by inhibiting excessive ERS [18]. So we hypothesized that oxidative stress and ERS play important roles in the pathogenesis of MI. And this study was therefore undertaken to investigate whether EPC can protect myocardium against MI by suppressing oxidative stress and excessive ERS, the key proteins—GRP78 and CHOP.

## 2. Materials and Methods

### 2.1. EPC Preparation

EPC was provided by Institute of Chinese Materia Medica, China Academy of Chinese Medical Sciences. The main components were shown in Table 1, measured by high performance liquid chromatogram (HPLC) method. 

### 2.2. Animals and Experimental Protocol

A total of 100 male Wistar rats weighing 180 ± 20 g were purchased from the Institute of Laboratory Animal Sciences, Chinese Academy of Medical Sciences (Certificate no. SCXK Beijing 2005-0013). The protocol was approved by the animal care and ethics committee of the China Academy of Chinese Medical Sciences. Sham group comprised 10 randomly selected rats, and the remainder was randomly divided into 5 groups, namely, control group, metoprolol group, low-dose EPC group, moderate-dose EPC group, and high-dose EPC group, with 18 rats in each group. The left anterior descending (LAD) coronary artery was ligated in the 5 groups to establish MI model according to Olivetti's methods as described before [19, 20]. The rats were anesthetized by intraperitoneal injection of urethane solution (20%) at a dose of 0.6 mL/kg. The rats in sham group did not undergo ligation. Of the surviving rats, metoprolol (AstraZeneca Pharmaceutical Co., Ltd., batch no.: 1012055), EPC were administered to metoprolol group (9 mg/kg), low-dose EPC group (0.54 g/kg), moderate-dose EPC group (1.08 g/kg), and high-dose EPC group (2.16 g/kg) by gastrogavage, respectively, once every 24 h for two weeks, and an equal volume of normal saline was given to sham group and control group [21]. One hour after the last administration, the blood samples were collected from the abdominal aorta of rats and kept in a red tube biochemical procoagulant at room temperature for 60 min. The serum was separated by low-speed centrifugation and then was stored at −80°C for use. The myocardial tissues below the ligature were stored in liquid nitrogen for Western blotting analysis.

### 2.3. Enzyme-Linked Immunosorbent Assay

The serum levels of malondialdehyde (MDA), SOD, and 8-iso-prostaglandin F2*α* (8-iso-PGF2*α*) were detected using enzyme-linked immunosorbent assay (ELISA) according to the manufacturer's instructions. The ELISA kits were provided by Sino-American Biotechnology Co., Ltd. (Wuhan, China). A Multiskan type 3 microplate reader (Thermo Scientific) was used for detection.

### 2.4. Quantitative Real-Time Polymerase Chain Reaction (PCR)

Total mRNA was extracted using Trizol reagent (Invitrogen) according to the manufacturer's protocol. The mRNA was reverse transcribed to cDNA using M-MLV reverse transcriptase PCR Kit (TaKaRa). The primer sets for GRP78 (forward 5′-CCTGGTTCTGCTTGATGTGT-3′ and reverse 5′-TCGTTCACCTTCGTAGACCTT-3′), CHOP (forward 5′-CCAGGAAACGAAGAGGAAGA-3′ and reverse 5′-GGTGCTTGTGACCTCTGCT-3′), and glyceraldehydes phosphate dehydrogenase (GAPDH) (forward 5′-CAACTCCCTCAAGATTGTCAGCAA-3′ and reverse 5′-GGCATGGACTGTGGTCATGA-3′) were synthesized by Shanghai Sangon Biotech Co., Ltd. PCR amplification of GRP78, CHOP, and GAPDH cDNAs was performed with 1.5 *μ*L cDNA in the same parameters. The reverse transcription PCR and analysis were performed using the ABI PRISM 7500 sequence detection system. Reactions were run for optimal cycles with predenaturalization at 94°C for 15 min; denaturation, annealing, and extension at 94°C for 15 s, 60°C for 34 s, 72°C for 15 s and repeated for 40 cycles; and lastly extension at 72°C for 10 min. The housekeeping gene GAPDH was used for internal control. The 2^−ΔΔCT^ method [22] was used to analyze the relative changes in gene expression. 

### 2.5. Western Blotting

The myocardium tissues were homogenized and lysed in lysis buffer. Proteins were separated by sodium dodecyl sulfate-polyacrylamide gel electrophoresis (SDS-PAGE) and transferred to a polyvinylidene difluoride (PVDF) membrane. The blots were then incubated with the primary antibody against GRP78 (Abcam, USA) and CHOP (Cell Signaling Technology, USA) at 4°C overnight, and then the membrane was incubated with appropriate secondary antibody. After washing, membranes were exposed to X-ray film. The staining was quantified by scanning the films and the band density was determined with Image-Pro Plus software.

### 2.6. Statistical Analysis

All data from at least 9 (ELISA results) or 5 (real-time Quantitative PCR and Western blotting analysis) independent experiments were expressed as means ± standard deviation (SD). One-way analysis of variance (ANOVA) was carried out for the comparison of means. All statistical analyses were performed with SPSS version 11.0, and *P* values of less than 0.05 were considered to be statistically significant.

## 3. Results

### 3.1. General Condition

All the survived rats underwent operation exhibited normal physical appearance and behavior during the gavage period of different drugs. The survival outcome after LAD ligation is presented in Table 2.

### 3.2. Expressions of MDA, SOD, and 8-Iso-PGF2*α* in Serum

**Figure 2:**
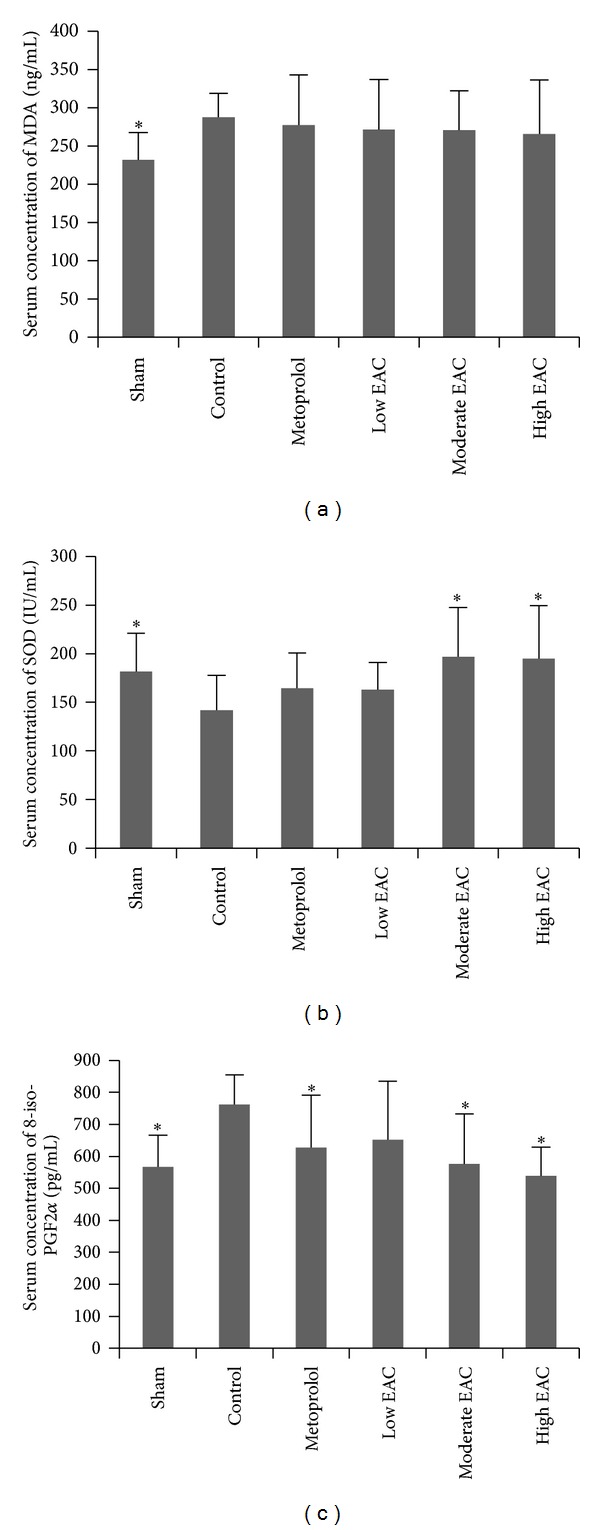
Serum concentration of MDA (a), SOD (b), and 8-iso-PGF2*α* (c). The error bars denote SD (*P < 0.05 compared with control group).

The serum concentrations of MDA, SOD, and 8-iso-PGF2*α* are shown in Figure 2. The serum MDA and 8-iso-PGF2*α* levels in control group were significantly increased, while the serum SOD level decreased, compared to sham group (*P* < 0.05). Moderate-to-high dose EPC increased SOD, decreased 8-iso-PGF2*α*, and metoprolol also decreased 8-iso-PGF2*α*, when, respectively, compared with control group (*P* < 0.05). 

### 3.3. EPC Reduces GRP78 and CHOP mRNA Expressions in Infarcted Myocardium

**Figure 3:**
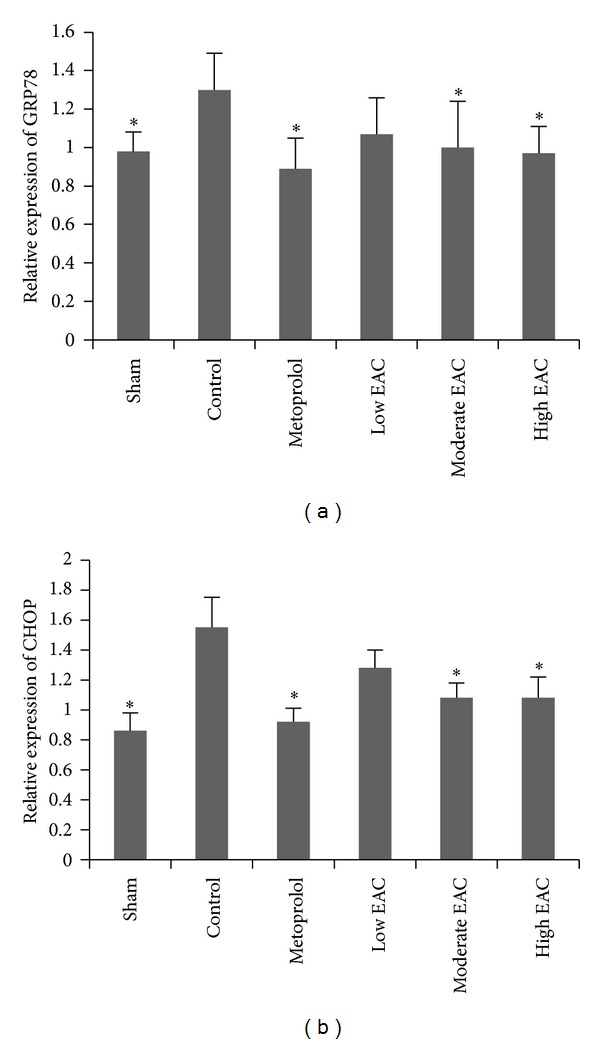
Expressions of GRP78 and CHOP mRNA in infarcted myocardium. The gene expressions of GRP78 and CHOP were determined by quantitative real-time PCR. GAPDH was used as a control reference. The error bars denote SD (*P < 0.05 compared with control group; *n* = 6).

Alterations in mRNA expression of GRP78 and CHOP in infarcted myocardium were detected by quantitative real-time PCR. Compared with sham group, the gene expression of GRP78 and CHOP increased after experimental AMI (*P* < 0.05). Metoprolol and moderate-to-high dose EPC significantly reduced the mRNA expression of GRP78 and CHOP when compared to that of control group (*P* < 0.05). The results are shown in Figure 3.

### 3.4. EPC Decreases GRP78 and CHOP Protein Expressions in Infarcted Myocardium

**Figure 4:**
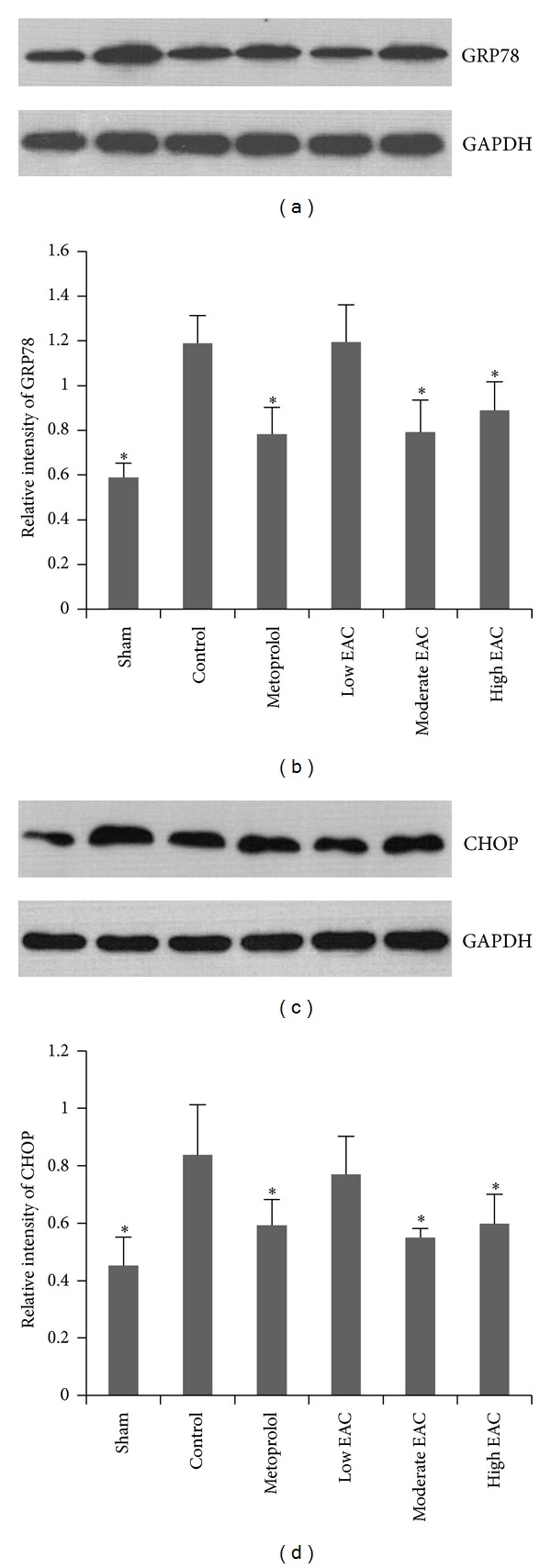
Expressions of GRP78 and CHOP protein in infarcted myocardium. The expressions of GRP78 and CHOP protein in infarcted myocardium were performed by Western blotting ((a) and (c)). Quantification of protein expressions were shown in (b) and (d). The error bars denote SD (*P < 0.05 compared with control group; *n* = 6).

Alterations in protein expression of GRP78 and CHOP in infarcted myocardium were detected by Western blotting. As seen in Figure 4, the protein expression of GRP78 and CHOP increased after experimental AMI (*P* < 0.05). Compared with control group, metoprolol and moderate-to-high dose EPC significantly decreased the protein expression of GRP78 and CHOP (*P* < 0.05).

## 4. Discussion

In the setting of AMI, ROS has been indicated playing a significant role in tissue necrosis and ischemia-reperfusion injury [23, 24]. Several pathways exist to protect against damage induced by ROS, with those best characterized in the heart being the superoxide dismutase. Overexpression of SOD has been shown to reduce infarct size in mice, which supports the contention that SOD is a major defense mechanism against ROS and a critical determinant in the tolerance of the heart to oxidative stress [25]. One method to quantify oxidative injury is to measure lipid peroxidation. MDA, one of the end-products of lipid peroxidation driven by ROS, can contribute significantly to the oxidative damage of proteins as it occurs under conditions of oxidative stress in age-related diseases and ischemic heart disease [26, 27]. Quantification of 8-iso-PGF2*α* derived from the nonenzymatic oxidation of arachidonic acid provides an accurate assessment of oxidative stress both in vitro and in vivo [28, 29], which was also identified as an independent and cumulative risk marker of coronary heart disease [30]. In the present study, the expressions of MDA and 8-iso-PGF2*α* in control group were increased compared to sham group, while the expression of SOD decreased, which indicats that MI conditions induce oxidative stress.

Perturbations of ER homeostasis affect protein folding and cause ERS. MI conditions induce accumulation of unfolding or misfolding proteins within the ER. ER can sense the stress and then respond to it through translational attenuation, upregulation of the genes for ER chaperones and related proteins, and degradation of unfolded proteins by a quality-control system [31]. GRP78, belonging to the heat shock protein 70 group and widely used as a marker for ERS, plays an important role in many cellular processes, which can contribute to the repair of unfolded proteins [32]. One important component of the ERS-mediated apoptosis pathway is CHOP, which encourages ROS production by depleting the cell of glutathione [31]. The results showed that both the gene and protein expressions of GRP78 and CHOP in control group were increased compared to sham group, indicating that MI conditions also induce ERS. Therefore, MI conditions induce both excessive ERS and oxidative stress.

Beta-blockers have been used extensively in the last 40 years after AMI as part of primary therapy and in secondary prevention. Metoprolol, a Beta-blocker, as a cornerstone in the therapy of the postinfarct heart, has an important effect on decreasing mortality in patients after AMI [33]. George et al. reported that metoprolol can significantly improve cardiac function, result in normalized ERS marker, and reduce DNA damage in a coronary embolization model of heart failure [34]. The aforesaid results showed that metoprolol downregulated the expressions of GRP78 and CHOP in myocardium subjected to MI, protecting the myocardium by attenuating ERS. Metoprolol also decreased 8-iso-PGF2*α* serum level so as to suppress oxidative stress invoked by MI. Therefore, metoprolol protect myocardium by suppressing excessive ERS and oxidative stress.

EPC, the extracts of *Panax quinquefolius* and *Corydalis tuber*, has been used for the treatment of ischemic cardiovascular diseases for years in clinic. *Panax quinquefolius *saponins and tetrahydropalmatine are the main components of EPC determined by HPLC method. Previous animal experiments and clinical trials have shown that *Panax quinquefolius *saponins have antioxidant effects, and its protective effects may be mostly attributed to scavenging H_2_O_2_ and hydroxyl radicals, enhancing the activities of superoxide dismutase and catalase, suppressing ROS-induced Jun N-terminal kinase activation [35–37]. Tetrahydropalmatine has been shown to have a protective effect against oxidative stress, which significantly reduced intracellular ROS formation and enhanced the production of intracellular antioxidants—SOD. Wang et al. reported that *Panax quinquefolius* saponins suppressed hypoxia-reoxygenation-induced excessive ERS, as evidenced by reduced caspase 12 activation and decreased GRP78, calreticulin, and CHOP [38]. Our findings presented here confirm and extend findings of the aforesaid works. EPC exhibited significant protective effects against oxidative stress injury in myocardium after MI by increasing SOD and decreasing 8-iso-PGF2*α*. Moderate-to-high dose EPC significantly decreased the mRNA and protein expressions of GRP78 and CHOP when compared with control group, indicating that EPC could alleviate injury of myocardium subjected to MI by suppressing excessive ERS. Based on our study, ERS and oxidative stress are potential therapeutic targets for human AMI. The beneficial effects of metoprolol on MI are mediated, at least in part, through the prevention of oxidative stress and ERS induced damage. EPC is an effective compound for treatment of MI by suppressing excessive ERS and oxidative stress, which provides experimental evidence for the clinical application of EPC.

## 5. Conclusions

Metoprolol and EPC protect the myocardium by attenuating oxidative stress and ERS in MI rats, highlighting the ERS pathways as potential therapeutic targets for MI. Further mechanistic study will be necessary to elucidate these interactions fully.

## Figures and Tables

**Table 1:** Quality evaluation of EPC.

Major constituent	Content (%)
Ginsenoside Rg1	0.11
Ginsenoside Re	1.88
Ginsenoside Rb1	5.30
Tetrahydropalmatine	0.07

**Table 2:** The outcome after LAD ligation.

Group	*N*	Dead rats (*n*)	Surviving rats (*n*)
Sham	10	0	10
Control	18	9	9
Metoprolol	18	6	12
Low EPC	18	9	9
Moderate EPC	18	7	11
High EPC	18	8	10
